# Acute Kidney Injury (AKI) in Severe Plasmodium falciparum Malaria Among Adult Sudanese Patients in Kosti Teaching Hospital, 2025

**DOI:** 10.7759/cureus.113534

**Published:** 2026-07-28

**Authors:** Ziryab Imad, Shazer Mubarak Mohamedkhair Mohamed, Mustafa Awad, Tarig Alhadi Madibbo Ahmed

**Affiliations:** 1 Internal Medicine, Haj El-Safi Teaching Hospital, Khartoum North, SDN; 2 Internal Medicine, University of Bahri, Khartoum, SDN; 3 Internal Medicine, University of El Imam El Mahdi, Khartoum, SDN; 4 Internal Medicine, Kosti Teaching Hospital, Kosti, SDN; 5 Internal Medicine, Al Neelain University, Khartoum, SDN; 6 Medicine, University of Bahri, Khartoum, SDN

**Keywords:** acute kidney injury, clinical features, malaria, malaria clinical features, severity of malaria

## Abstract

Background

Acute kidney injury (AKI) is a frequent and serious complication of severe malaria (SM), contributing significantly to morbidity and mortality. In endemic regions like Sudan, AKI complicates the clinical course of SM, yet detailed demographic, clinical, and outcome data remain limited.

Aim

This study aimed to characterize the demographic profile, clinical features, management strategies, and outcomes of AKI in patients with SM in Sudan.

Methods

A cross-sectional analysis of 100 SM patients with AKI was conducted in Kosti Teaching Hospital from February to May 2025. Data on demographics, comorbidities, malaria complications, AKI severity (classified via Kidney Disease: Improving Global Outcomes (KDIGO) criteria), treatment regimens, contributing factors, interventions, and outcomes were collected from medical records. The statistical analysis was performed using SPSS (IBM SPSS Statistics for Windows, IBM Corp., Version 26.0, Armonk, NY).

Results

Patients were predominantly male (64 (64%)) with a mean age of 48 years (SD 22); 43 (43%) were under 40 years of age. Central Sudan residents comprised 67 (67%) of cases. Hyperparasitemia (92 (92%)), circulatory shock (58 (58%)), and prostration (44 (44%)) were frequent complications. Hypertension (36 (36%)) and diabetes (20 (20%)) were leading comorbidities. Artesunate was the primary treatment (68 (68%)). AKI severity included Stage 1 (44 (44%)), Stage 2 (28 (28%)), and Stage 3 (28 (28%)). Hypovolemia (95 (95%)) and sepsis (63 (63%)) were key contributors. Interventions included IV fluids (94 (94%)) and antibiotics (74 (74%)). Outcomes revealed full renal recovery in 55 (55%), partial recovery in 28 (28%), progression to chronic kidney disease in 13 (13%), and a four (4%) mortality rate.

Conclusion

SM-associated AKI in Sudan predominantly affects younger people as well as males and is frequently linked to hyperparasitemia, circulatory shock, and comorbid hypertension. Despite high rates of severe AKI, over half of patients achieve full renal recovery with appropriate treatment, though a notable proportion progress to chronic kidney disease or die.

## Introduction

Malaria remains one of the world's most prevalent infectious diseases and continues to pose a substantial public health challenge, particularly in developing countries. Globally, an estimated 229 million malaria cases and 409,000 malaria-related deaths occurred in 2019. Africa accounted for the vast majority of these cases, while children represented nearly 67% of all malaria deaths worldwide [1].

The disease is transmitted through the bite of an infected female *Anopheles* mosquito carrying *Plasmodium* species, allowing the parasite to enter the human bloodstream [2]. The clinical manifestations of malaria vary widely, ranging from asymptomatic infection to uncomplicated malaria, which typically presents with non-specific symptoms such as fever, headache, and myalgia. In more severe cases, patients may develop severe malaria (SM), a life-threatening condition characterized by complications including severe anemia, coma, acute kidney injury (AKI), and metabolic acidosis [3].

AKI is characterized by a sudden decline in renal function and is recognized as one of the most severe complications of *Plasmodium falciparum* malaria, with a well-established association with increased mortality. According to Kidney Disease: Improving Global Outcomes (KDIGO) criteria, AKI is diagnosed by one of the following: 1) an increase in serum creatinine of at least 0.3 mg/dL within 48 hours, 2) an increase in serum creatinine to at least 1.5 times the baseline value within the preceding seven days, or 3) a reduction in urine output to less than 0.5 mL/kg/hour for at least six hours [4]. The development of AKI is multifactorial and may result from prerenal causes such as hypovolemia or impaired renal perfusion; postrenal causes including urinary tract obstruction; or intrinsic renal causes such as nephrotoxic injury, infection, and inflammation [5]. Before the adoption of the KDIGO consensus criteria, the true burden of malaria-associated AKI in both adults and children was substantially underestimated [6,7]. More recent investigations applying the KDIGO definition have reported AKI prevalence rates of 20-40% among both adult and pediatric patients with SM, while incidences as high as 59% have been documented in children [8-13]. Although AKI occurs with comparable frequency in adults and children with SM, the overall disease burden is greater among children because they constitute the majority of SM cases in endemic regions [14].

In Sudan, malaria continues to be a major public health problem, with nearly three-quarters of the population living in areas at risk of infection [15]. Although *Plasmodium falciparum* is the predominant endemic species, malaria-associated AKI remains inadequately investigated. Available evidence indicates that AKI develops in approximately 39% of patients with SM admitted to intensive care units and is associated with a striking mortality rate of 87.8% [16]. Furthermore, most current evidence regarding the epidemiology, risk factors, underlying mechanisms, and clinical outcomes of AKI in SM originates from studies conducted in Asia and other non-African settings, leaving significant gaps in knowledge regarding the Sudanese adult population.

Justification/rationale

The study focuses on the significant issue of AKI in adult Sudanese patients suffering from SM, highlighting its prevalence as a critical determinant of poor health outcomes. Given the high incidence of acquired AKI in Sudan, there is an urgent need to address this complication within the country's healthcare system, particularly in areas heavily impacted by malaria such as Kosti. The research aims to investigate the frequency, determinants, and outcomes of AKI among SM patients.

## Materials and methods

Study design/setting

This is a descriptive observational hospital-based study; it was conducted in the Internal Medicine Department at Kosti Teaching Hospital, White Nile State, between February and May 2025.

Study population

Inclusion Criteria

Adult patients (≥18 years); patients with SM according to the Sudan Malaria Diagnosis and Treatment Protocol [15]; patients with confirmed *Plasmodium falciparum* infection via rapid diagnostic test (RDT) or microscopy; and patients with complete medical records were included in the study.

Exclusion Criteria

Those who were excluded were those with *Plasmodium vivax* infection, pre-existing chronic kidney disease (CKD) or end-stage renal disease (ESRD), and recent nephrotoxic drug use or non-malarial sepsis.

Sample size and sampling technique

The authors followed a total coverage approach as a sampling method, where all SM patients diagnosed with AKI who fulfilled the inclusion criteria during the determined study period were included. The total number of samples obtained during the study period was 100 patients.

Data collection tools and methods

Data collection was carried out by the principal investigator through a structured questionnaire composed of demographic data, medical history, clinical presentations, and AKI.

SM was assessed using the WHO malaria severity assessment form, and AKI was assessed by KDIGO criteria, including serum creatinine and urine output.

Data analysis

Data were entered and cleaned of errors, repetition, duplication, and missing data using Microsoft Excel 2019 (Microsoft® Corp., Redmond, WA) and analyzed using SPSS (IBM SPSS Statistics for Windows, IBM Corp., Version 26.0, Armonk, NY). The analysis used descriptive statistics, which consisted of frequency tables and figures. Data were presented in the form of tables and figures.

Ethical consideration

Ethical approval was obtained from the Ministry of Health, White Nile State-Kosti Teaching Hospital. Data were used anonymously by using identity numbers instead of names in order to protect patients' confidentiality. No reference to any individual participant was made in study reports. Informed written consent was obtained from each participant, and the participants were aware of their ability to withdraw from the study at any point in time.

## Results

Table 1 summarizes the demographic characteristics of the 100 SM patients with AKI included in the study. The majority were male (64 (64%)), while females comprised 36 (36%). The mean age was 48 years (SD 22), and most patients were aged under 40 years (43 (43%)). The majority of the patients resided in Central Sudan (67 (67%)), followed by Khartoum (29 (29%)), with smaller proportions from Western and Southern Sudan. Regarding occupation, workers represented 24 (24%), followed by housewives (22 (22%)), students (21 (21%)), retired individuals (19 (19%)), and employees (14 (14%)).

**Table 1:** The demographic characteristics of severe malaria patients with acute kidney injury (N = 100)

	N	%
Gender		
Male	64	64.0
Female	36	36.0
Age (years): mean ± SD (48 ± 22)		
18-39	43	43.0
40-60	23	23.0
>60	34	34.0
Residence		
Central Sudan	67	67.0
Khartoum	29	29.0
Western Sudan	3	3.0
Southern Sudan	1	1.0
Occupation		
Worker	24	24.0
Housewife	22	22.0
Student	21	21.0
Retired	19	19.0
Employee	14	14.0

**Figure 1:**
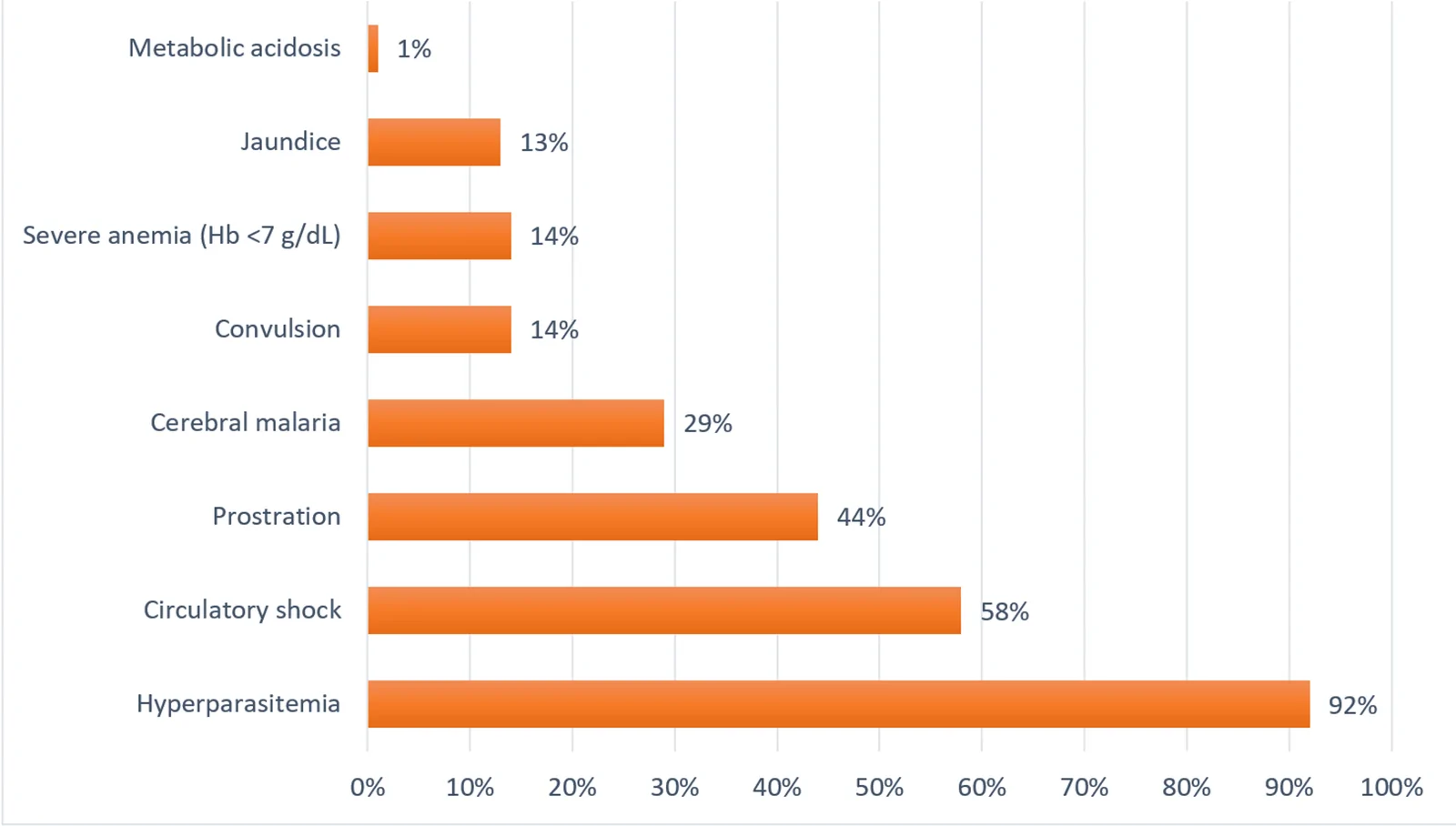
The clinical features of severe malaria patients with acute kidney injury (N = 100)

Hyperparasitemia was the most common SM manifestation, present in 92 (92%) of cases, followed by circulatory shock in 58 (58%), prostration in 44 (44%), and cerebral malaria in 29 (29%). Less frequent features included convulsions and severe anemia, each occurring in 14 (14%) of patients, jaundice in 13 (13%), and metabolic acidosis in one (1%) (Figure 1).

**Figure 2:**
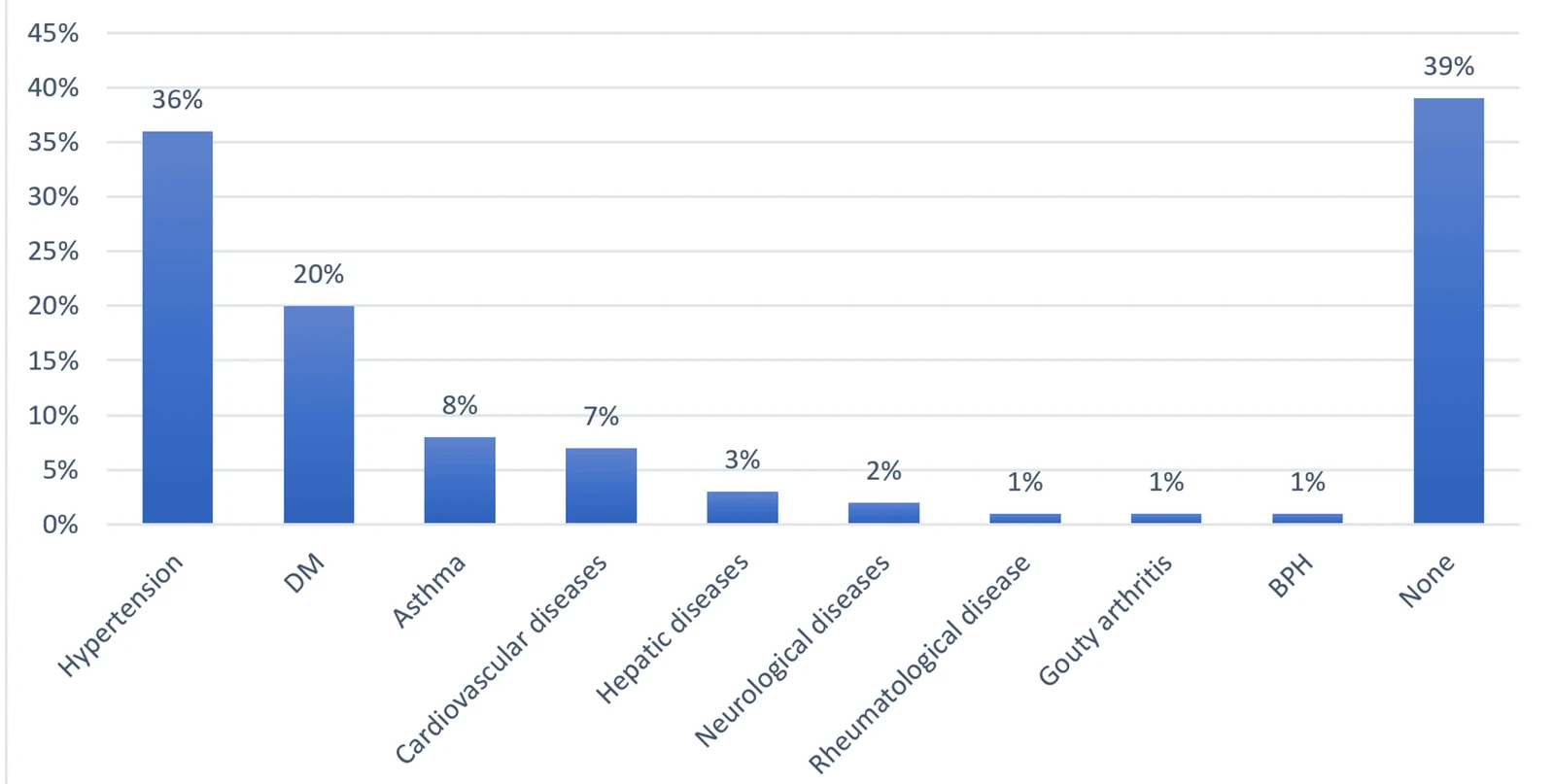
The comorbidities among severe malaria patients with acute kidney injury (N = 100)

Hypertension was the most prevalent comorbidity, affecting 36 (36%) of patients, followed by diabetes mellitus in 20 (20%), asthma in eight (8%), and cardiovascular diseases in seven (7%). Other comorbidities, including hepatic, neurological, and rheumatological diseases; gouty arthritis; and benign prostatic hyperplasia, were less common, while 39 (39%) of patients had no comorbidities (Figure 2).

The majority of patients (82 (82%)) had experienced two or more malaria episodes in the previous year, while 18 (18%) reported only one episode.

**Figure 3:**
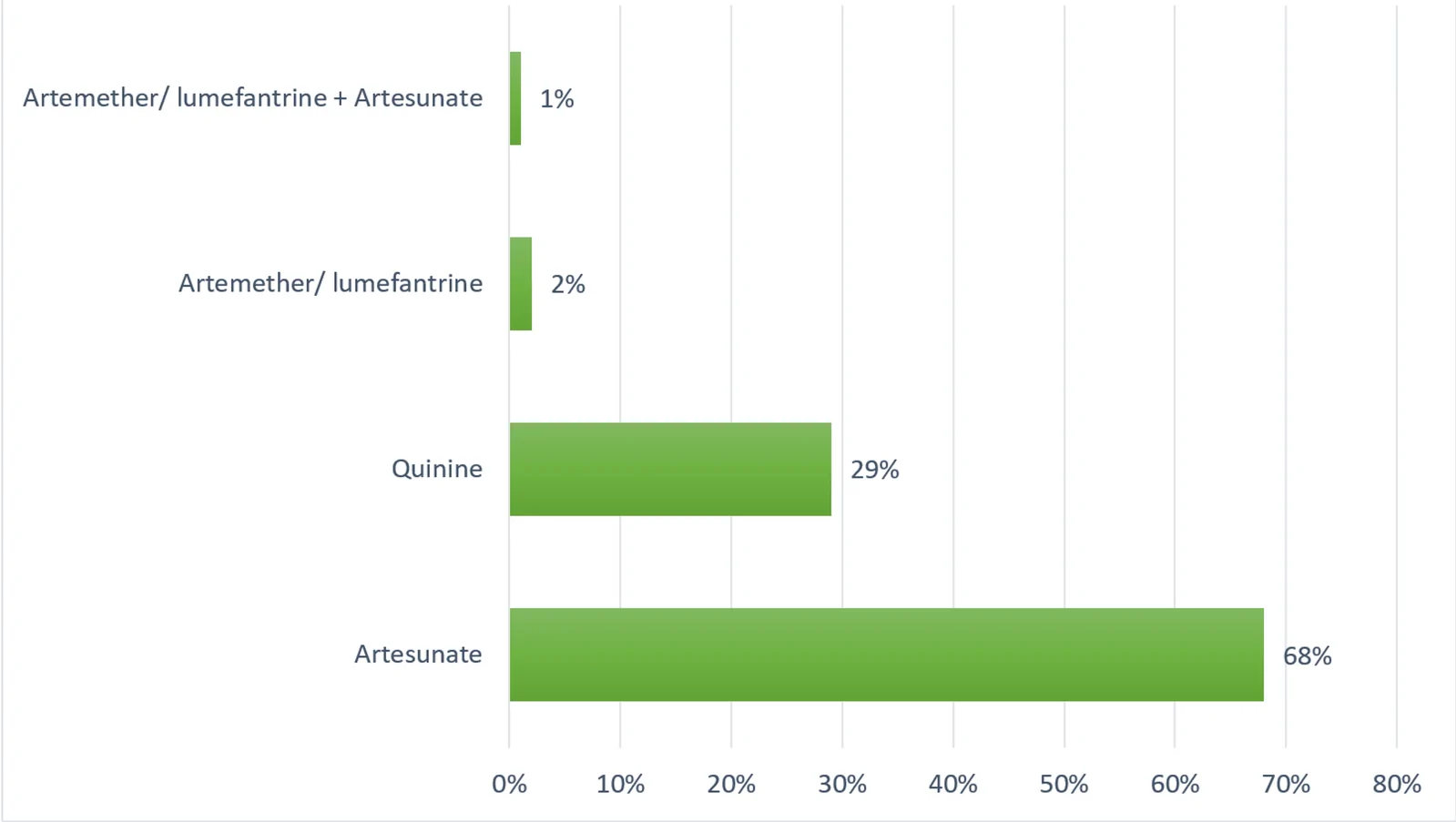
Antimalarial treatment received by severe malaria patients with acute kidney injury (N = 100)

Artesunate was the most commonly used treatment, administered to 68 (68%) of patients, followed by quinine in 29 (29%). Artemether/lumefantrine was used in two (2%) of cases, while a combination of artemether/lumefantrine and artesunate was used in one (1%) of patients (Figure 3).

The mean serum creatinine level was 3.2 mg/dL (SD 2.7), with 49 (49%) of patients having levels ≤2 mg/dL and 51 (51%) above this threshold. The average 24-hour urine output was 1,426 mL (SD 1,037). The mean hemoglobin concentration was 9.8 g/dL (SD 2.7), with 10 (10%) of patients having hemoglobin levels <6 g/dL, 34 (34%) between 6 and 9 g/dL, and 56 (56%) ≥10 g/dL. The mean leukocyte count was 9.1 × 10⁹/L (SD 2.7), with 52 (52%) of patients having counts ≤8 × 10⁹/L and 48 (48%) >8 × 10⁹/L. Platelet counts averaged 169 × 10⁹/L (SD 105), with 52 (52%) of patients having platelet counts <140 × 10⁹/L. The mean C-reactive protein (CRP) level was 23 mg/L (SD 18), with 68 (68%) of patients having CRP levels ≥10 mg/L (Table 2).

**Table 2:** The lab findings of severe malaria patients with acute kidney injury (N = 100)

	N	%
Cr (mg/dL); mean ± SD	3.2 ± 2.7	
≤2	49	49.0
>2	51	51.0
Urine output in 24 hours (mL); mean ± SD	1426 ± 1037	
Hb (g/dL); mean ± SD	9.8 ± 2.7	
<6	10	10.0
9-6	34	34.0
≥10	56	56.0
Leucocyte count (×10⁹/L); mean ± SD	9.1 ± 2.7	
≤8	52	52.0
>8	48	48.0
Platelets count (×10⁹/L); mean ± SD	169 ± 105	
<140	52	52.0
≥140	48	48.0
CRP (mg/L); mean ± SD	23 ± 18	
<10	32	32.0
≥10	68	68.0

**Figure 4:**
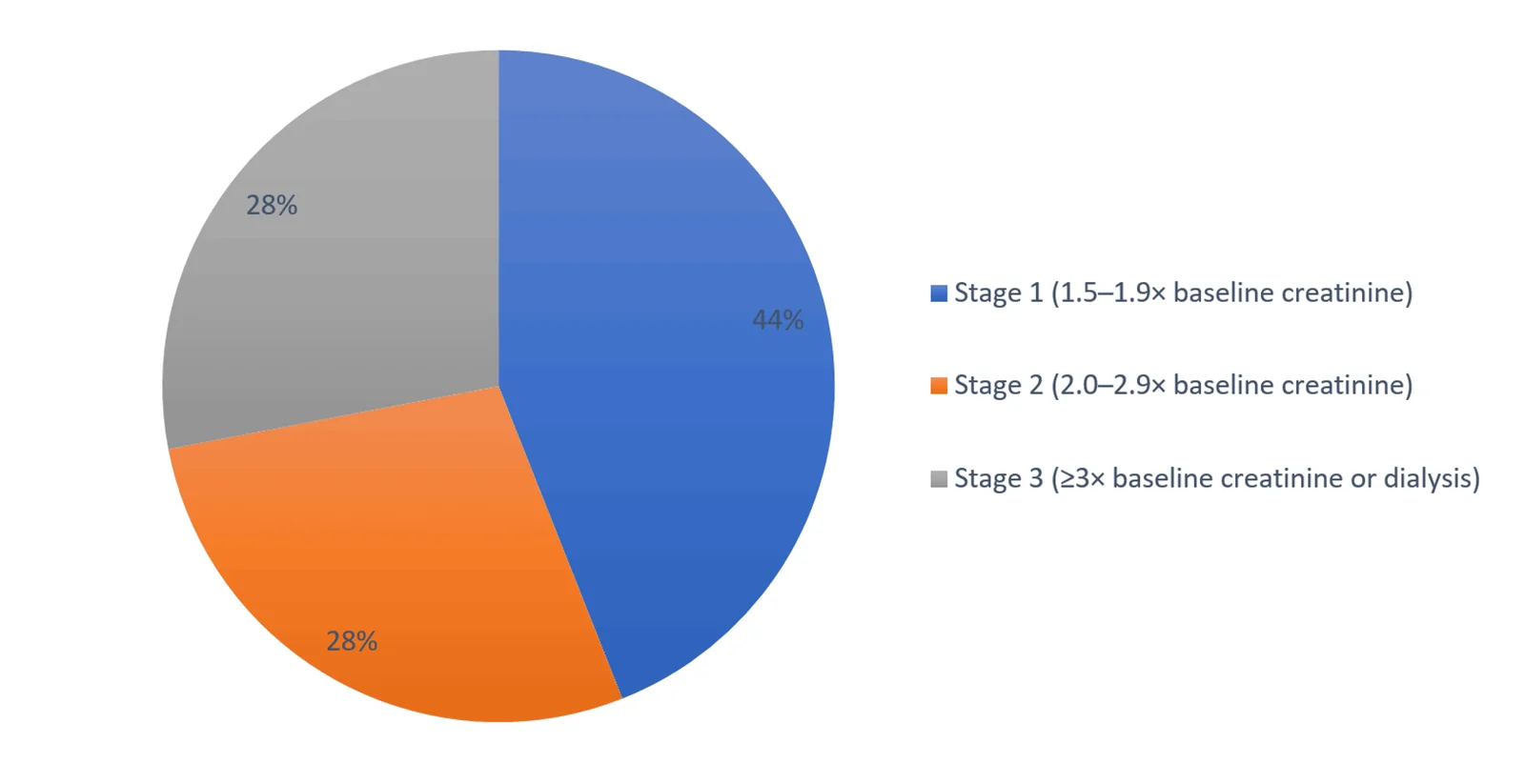
Acute kidney injury diagnosis based on Kidney Disease: Improving Global Outcomes (KDIGO) criteria among severe malaria patients (N = 100)

Figure 4 illustrates the distribution of AKI severity based on the KDIGO criteria. Stage 1 (1.5-1.9 times baseline serum creatinine) was observed in 44 (44%) of patients, Stage 2 (2.0-2.9 times baseline) in 28 (28%), and Stage 3 (≥3 times baseline serum creatinine or requiring dialysis) in 28 (28%).

The mean duration was six days (SD 4), with 38 (38%) developing AKI within one to three days, 49 (49%) within four to seven days, and 13 (13%) after more than seven days (Table 3).

**Table 3:** The time from malaria diagnosis to acute kidney injury onset among severe malaria patients with acute kidney injury (N = 100)

	N	%
Time from malaria diagnosis to AKI onset (days); mean ± SD	6 ± 4	
1-3	38	38.0
4-7	49	49.0
>7	13	13.0

**Figure 5:**
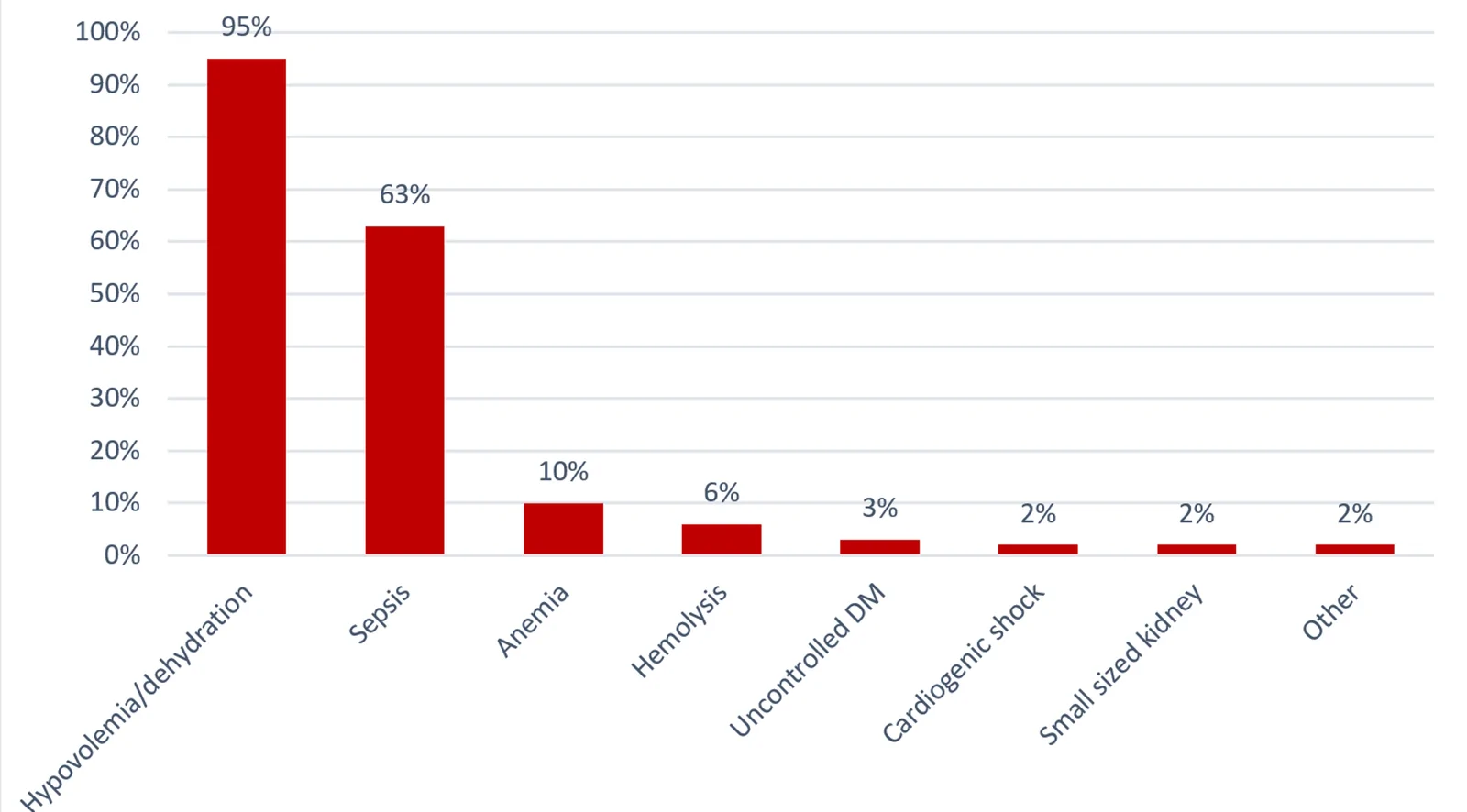
Suspected contributing factors to acute kidney injury among severe malaria patients (N = 100)

Figure 5 identifies suspected contributing factors to AKI. Hypovolemia or dehydration was suspected in 95 (95%) of cases, sepsis in 63 (63%), anemia in 10 (10%), and hemolysis in six (6%). Less common factors included uncontrolled diabetes mellitus, cardiogenic shock, small-sized kidneys, and other causes.

**Figure 6:**
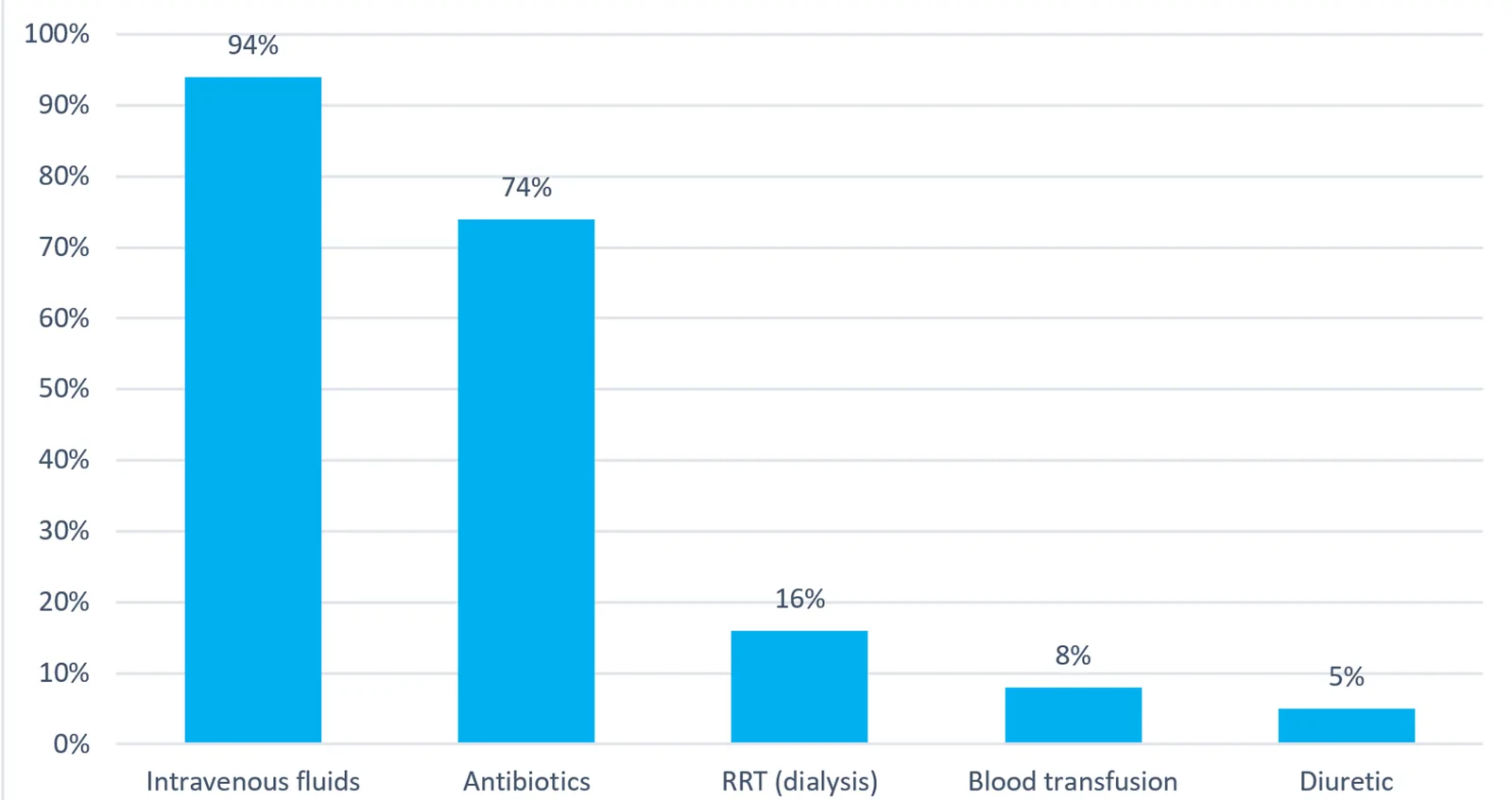
Interventions provided for acute kidney injury among severe malaria patients (N = 100)

Figure 6 summarizes the interventions provided for AKI. Intravenous fluids were administered to 94 (94%) of patients, antibiotics to 74 (74%), renal replacement therapy (dialysis) to 16 (16%), blood transfusion to eight (8%), and diuretics to five (5%).

**Figure 7:**
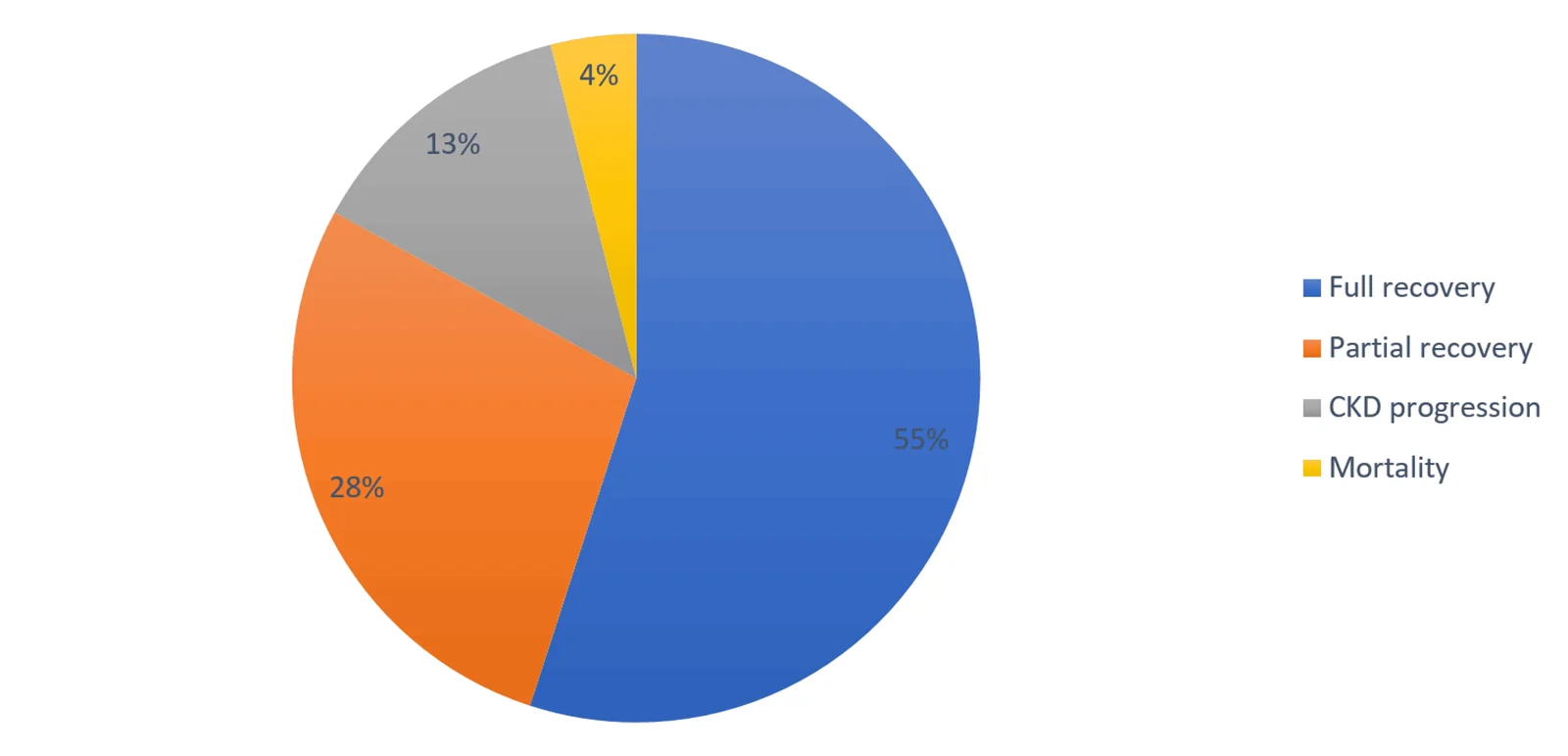
The outcome at discharge of severe malaria patients with acute kidney injury (N = 100)

In terms of outcomes, full recovery of renal function was achieved in 55 (55%) of patients, representing a favorable outcome for just over half of the study population. Partial recovery was observed in 28 (28%) of patients, indicating persistent renal dysfunction at the time of discharge. Progression to chronic kidney disease occurred in 13 (13%) of patients, representing a significant long-term complication. The mortality rate was 4 (4%), reflecting the life-threatening nature of severe malaria with acute kidney injury despite medical interventions (Figure 7).

## Discussion

The findings of this study provide critical insights into the clinical and demographic landscape of SM complicated by AKI in Sudan among 100 patients, highlighting both modifiable risk factors and opportunities for improving patient outcomes. The predominance of male patients (64%) aligns with global trends in SM burden, which may reflect occupational or behavioral exposures, such as outdoor work in endemic regions. The mean age of 48 years (SD 22) and the high proportion of patients under 40 years (43%) emphasize the vulnerability of younger, economically active populations to SM complications, with implications for public health prioritization in resource-limited settings. Our findings were consistent with prior studies by Muhamedhussein et al. and Umuhire et al., which indicated higher AKI risk among males and middle-aged adults in malaria-endemic regions [11,17]. However, Afolayan et al. reported that AKI among SM patients was comparable between males and females [8]. The majority residing in Central Sudan and Khartoum reflects regional malaria burden distribution.

The overwhelming prevalence of hyperparasitemia (92%) as a key feature emphasizes its role in driving SM pathogenesis, likely exacerbating systemic inflammation and endothelial dysfunction, which contribute to AKI. The high frequency of circulatory shock (58%) and prostration (44%) further supports the hypothesis that hemodynamic instability and tissue hypoperfusion are central to AKI development in this cohort. The relatively low rates of cerebral malaria (29%) and severe anemia (14%) contrast with some regional studies, like Afolayan et al., who reported the rates of cerebral malaria and severe anemia were 38.8% and 50.6%, respectively [8], suggesting variability in malaria complications across populations or differences in diagnostic criteria.

Comorbidities, such as hypertension (36%) and diabetes mellitus (20%), may predispose patients to AKI by impairing renal autoregulation and increasing susceptibility to ischemic injury. Our findings were in agreement with the study of Muhamedhussein et al. [11]. The finding that 39% of patients had no comorbidities highlights the direct nephrotoxic potential of *Plasmodium falciparum* infection itself, independent of preexisting conditions.

The distribution of AKI severity by KDIGO criteria - 44% Stage 1, 28% Stage 2, and 28% Stage 3 - parallels findings from other cohorts where mild to severe AKI stages are common among SM patients. In the study of Conroy et al., most of the patients (51.8%) were AKI Stage 1 [9]. In addition, in the study of Saravu et al., out of 174 SM patients with AKI, 68% were AKI Stage 1 [18].

The mean duration of six days and the timing of AKI onset predominantly within the first week post-admission emphasize the acute nature of kidney injury in malaria and the critical window for intervention. However, the delayed AKI onset in 13% of patients (>7 days) suggests that ongoing monitoring beyond the acute phase is essential, particularly in resource-constrained settings where follow-up is challenging. In the study of Muhamedhussein et al., most of the malaria patients developed AKI at 48 hours [11]. In general, renal insufficiency in the setting of SM l infection commonly develops three to seven days after the onset of fever; serum creatinine typically improves in 17 ± 6 days [12,19].

Hypovolemia or dehydration (95%) and sepsis (63%) as major contributing factors highlight the multifactorial etiology of AKI in malaria, involving both parasite-mediated and host-related insults such as volume depletion and systemic infection. Correspondingly, in the article by Umuhire et al., the significant etiology associated with the development of AKI among SM patients was dehydration [17].

The high use of intravenous fluids (94%) and antibiotics (74%) reflects adherence to sepsis and AKI management guidelines [20]. However, the limited utilization of renal replacement therapy (16%) - despite 28% reaching Stage 3 AKI - may indicate barriers to dialysis access, potentially contributing to the 13% progression to CKD. The frequencies of dialysis in the studies of Muhamedhussein et al. and Koopmans et al. were 2% and 20.5%, respectively [11,12].

The favorable outcome of full renal recovery in 55% of patients is in line with the literature suggesting that timely diagnosis and management can result in substantial recovery [17,21]. However, the notable rates of partial recovery (28%) and progression to CKD (13%) highlight the potential for long-term renal impairment, underscoring the need for follow-up care. In the study, Assounga et al. found that up to 12% of patients developed chronic impairment in kidney function after malarial AKI [21].

The low mortality rate (4%) compared to other studies in similar settings could be attributed to the widespread use of artesunate (68%), which is associated with improved survival in SM. In the literature, the mortality rates, in-hospital deaths in particular, vary from 2% to 51% [20]. Our death rate was similar to the study of Afolayan et al. (4.8%) [8] and lower than that reported by Umuhire et al. (15%) [17].

Limitations

The study has several limitations. Firstly, it is a cross-sectional, single-center design that limits the generalizability of the findings and precludes establishing causal relationships. The relatively small sample size and reliance on hospital records may have introduced selection and information bias. Selection bias is another substantial issue because only patients with AKI were included. In the absence of a comparison group, determinants or predictors of AKI cannot be truly evaluated. CKD progression cannot be confidently diagnosed without follow-up beyond hospital discharge. Additionally, outcomes were assessed only until hospital discharge, preventing evaluation of long-term renal outcomes. Finally, the absence of multivariable analysis limited the identification of independent predictors of AKI severity and patient outcomes.

## Conclusions

AKI is a frequent and clinically significant complication of severe *Plasmodium falciparum* malaria among adult Sudanese patients. The condition was commonly associated with hyperparasitemia, hypovolemia, circulatory shock, and underlying comorbidities such as hypertension and diabetes mellitus. Most patients presented with Stage 1 AKI, although more than one-quarter had severe (Stage 3) disease. While the majority of patients achieved complete renal recovery following prompt management, a considerable proportion experienced persistent renal dysfunction, progressed to CKD, or died. These findings highlight the importance of early recognition, timely management of SM and its complications, and close renal monitoring to improve clinical outcomes and reduce the burden of malaria-associated AKI in Sudan.

## References

[1] (2025). World Malaria Report 2020. https://www.mmv.org/newsroom/news-resources-search/world-malaria-report-2020?gad_source=1&gad_campaignid=6609916219&gbraid=0AAAAAD9AoJrA5LNBQH5-Q4tJUrLzpA-Ky&gclid=Cj0KCQjwjIPSBhCCARIsABGyK7topwSTVdfVDoi5krFdTKXdxtHWGu8z_Xez6LCErJScWRLCwYUA13caAiSdEALw_wcB.

[2] Coban C, Lee MS, Ishii KJ (2018). Tissue-specific immunopathology during malaria infection. Nat Rev Immunol.

[3] Phillips MA, Burrows JN, Manyando C, van Huijsduijnen RH, Van Voorhis WC, Wells TN (2017). Malaria. Nat Rev Dis Primers.

[4] Makris K, Spanou L (2016). Acute kidney injury: definition, pathophysiology, and clinical phenotypes. Clin Biochem Rev.

[5] Hertzberg D, Rydén L, Pickering JW, Sartipy U, Holzmann MJ (2017). Acute kidney injury-an overview of diagnostic methods and clinical management. Clin Kidney J.

[6] Plewes K, Turner GD, Dondorp AM (2018). Pathophysiology, clinical presentation, and treatment of coma and acute kidney injury complicating falciparum malaria. Curr Opin Infect Dis.

[7] Oshomah-Bello EO, Esezobor CI, Solarin AU, Njokanma FO (2020). Acute kidney injury in children with severe malaria is common and associated with adverse hospital outcomes. J Trop Pediatr.

[8] Afolayan FM, Adedoyin OT, Abdulkadir MB, Ibrahim OR, Biliaminu SA, Mokuolu OA, Ojuawo A (2020). Acute kidney injuries in children with severe malaria: a comparative study of diagnostic criteria based on serum cystatin C and creatinine levels. Sultan Qaboos Univ Med J.

[9] Conroy AL, Hawkes M, Elphinstone RE (2016). Acute kidney injury is common in pediatric severe malaria and is associated with increased mortality. Open Forum Infect Dis.

[10] Conroy AL, Opoka RO, Bangirana P (2019). Acute kidney injury is associated with impaired cognition and chronic kidney disease in a prospective cohort of children with severe malaria. BMC Med.

[11] Muhamedhussein MS, Ghosh S, Khanbhai K, Maganga E, Nagri Z, Manji M (2019). Prevalence and factors associated with acute kidney injury among malaria patients in Dar es Salaam: a cross-sectional study. Malar Res Treat.

[12] Koopmans LC, van Wolfswinkel ME, Hesselink DA, Hoorn EJ, Koelewijn R, van Hellemond JJ, van Genderen PJ (2015). Acute kidney injury in imported Plasmodium falciparum malaria. Malar J.

[13] Calice-Silva V, Sacomboio E, Raimann JG (2018). Diagnostic performance of salivary urea nitrogen dipstick to detect and monitor acute kidney disease in patients with malaria. Malar J.

[14] Katsoulis O, Georgiadou A, Cunnington AJ (2021). Immunopathology of acute kidney injury in severe malaria. Front Immunol.

[15] (2025). Sudan Malaria Diagnosis and Treatment Protocol 2017. https://reliefweb.int/report/sudan/sudan-malaria-diagnosis-and-treatment-protocol-2017.

[16] Magboul SM, Osman B, Elnour AA (2020). The incidence, risk factors, and outcomes of acute kidney injury in the intensive care unit in Sudan. Int J Clin Pharm.

[17] Umuhire L, Dushimiyimana V, Nkuranyabahizi M (2024). Factors associated with acute kidney injury and outcomes in patients with malaria in a district hospital in Rwanda. Afr Health Sci.

[18] Saravu K, Rishikesh K, Parikh CR (2014). Risk factors and outcomes stratified by severity of acute kidney injury in malaria. PLoS One.

[19] Kamath N, Iyengar A (2018). Infections and the kidney: a tale from the tropics. Pediatr Nephrol.

[20] Brown DD, Solomon S, Lerner D, Del Rio M (2020). Malaria and acute kidney injury. Pediatr Nephrol.

[21] Assounga AG, Assambo-Kieli C, Mafoua A, Moyen G, Nzingoula S (2000). Etiology and outcome of acute renal failure in children in congo-brazzaville. Saudi J Kidney Dis Transpl.

